# Citizen science: Development of a low-cost magnetometer system for a coordinated space weather monitoring

**DOI:** 10.1016/j.ohx.2024.e00580

**Published:** 2024-09-07

**Authors:** Hyomin Kim, David Witten, Julius Madey, Nathaniel Frissell, John Gibbons, William Engelke, Anderson Liddle, Nicholas Muscolino, Joseph Visone, Zhaoshu Cao

**Affiliations:** a Department of Physics, New Jersey Institute of Technology, Newark, NJ, United States of America; b HamSCI Community, United States of America; c HamSCI Community (deceased), United States of America; d Department of Physics and Engineering, University of Scranton, Scranton, PA, United States of America; e Case School of Engineering, Case Western Reserve University, Cleveland, OH, United States of America; f College of Engineering, University of Alabama, Tuscaloosa, AL, United States of America; g Department of Computer Science, New Jersey Institute of Technology, now at Department of Mathematical Sciences, New Jersey Institute of Technology, Newark, NJ, United States of America

**Keywords:** Magnetometer, Space weather, Citizen science, Ionosphere, HF communication

## Abstract

As part of Ham Radio Science Citizen Investigation (HamSCI) Personal Space Weather Station (PSWS) project, a low-cost, commercial off-the-shelf magnetometer has been developed to provide quantitative and qualitative measurements of the geospace environment from the ground for both scientific and operational purposes at a cost that will allow for crowd-sourced data contributions. The PSWS magnetometers employ a magneto-inductive sensor technology to record three-axis magnetic field variations with a field resolution of ∼3 nT at a 1 Hz sample rate. The measurement range of the sensor is $\pm 1.1\times 10^{6}$ nT) and is valid over a temperature range of −40 °C to +85 °C. Data from the PSWS network will combine these magnetometer measurements with high frequency (HF, 3–30 MHz) radio observations to monitor large-scale current systems and ionospheric disturbances due to drivers from both space and the atmosphere. A densely-spaced magnetometer array, once established, will demonstrate their space weather monitoring capability to an unprecedented spatial extent. Magnetic field data obtained by the magnetometers installed at various locations in the US are presented and compared with the existing magnetometers nearby, demonstrating that the performance is very adequate for scientific investigations.

## Graphical abstract

**Figure:**
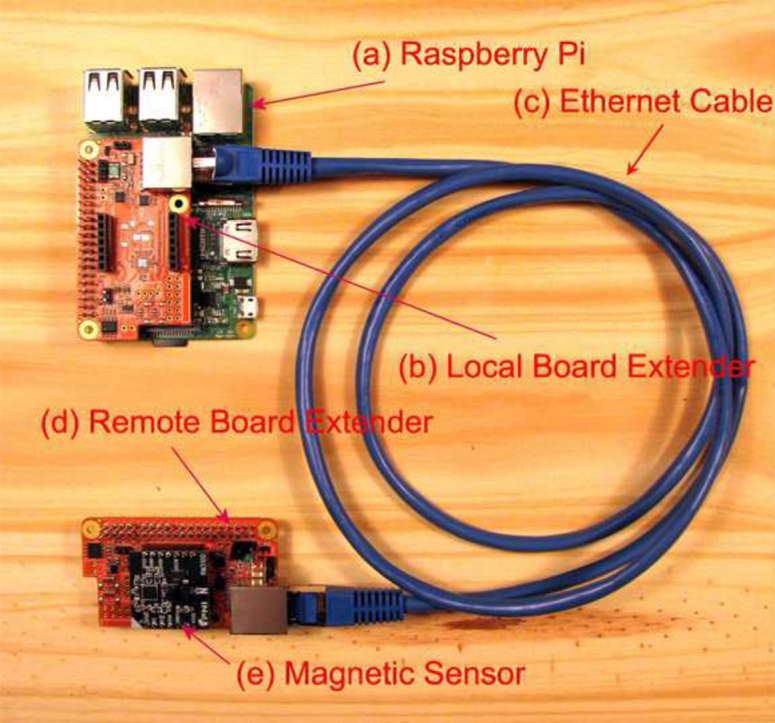

## Specifications table

**Table:** 

Hardware name	Magneto-inductive type magnetometer

Subject area	•Engineering and material science •Distributed low-cost sensors for geospace environment •Educational tools and open source alternatives to existing infrastructure

Hardware type	Field measurements and sensors

Closest commercial analog	An alternative technology is a “fluxgate” type sensor.

Open source license	The documentation is covered by the open source copyright requirements for CC by 4.0. Hardware is covered by the TAPR (Tucson Amateur Packet Radio) Open Hardware license. All supporting software is covered by the Gnu Public License 3.0.

Cost of hardware	Complete partially assembled kits of an equivalent design (local + remote) are currently available from the TAPR Webstore for $120.00.

Source file repository	Source code for the hardware is available for public download at: https://github.com/HamSCI/PSWS-Magnetometer. Source code for the software and documentation is available for public download at: https://github.com/HamSCI/rm3100-runMag. The design files and bill of materials are available at http://dx.doi.org/10.5281/zenodo.10289503.

OSHWA certification UID *(OPTIONAL)*	This has been applied for and is undergoing review.

## 1 Hardware in context

As part of a citizen-science project called Ham Radio Science Citizen Investigation (HamSCI) [1] funded by National Science Foundation (NSF) to investigate the effect of the solar–terrestrial environment to human technologies (“space weather”), we aim to create a small, multi-instrument system that can make ground-based measurements of the space environment called the “Personal Space Weather Station (PSWS)” (see [2]). This project will not only be useful to the owner of the system, but also aggregated into a central database for space science and space weather research purposes. Initial work focuses on the development of a scientific-grade high frequency (HF) radio receiver (called GRAPE [2]) and a magnetometer (“HamSCI magnetometer”), as well as the necessary software and network infrastructure.

This paper reports the development and construction of PSWS magnetometers which measure magnetic field strength and direction to provide quantitative and qualitative measurements of the geospace environment from the ground for both scientific and operational purposes at a cost that will allow for crowd-sourced data contributions. The HamSCI PSWS magnetometer and this paper were developed using a citizen science/open science collaboration between professional researchers and volunteers, with plans for this system originally appearing on the HamSCI Ground Magnetometer Website [3].

The HamSCI mag employs a low-cost, commercial off-the-shelf, magneto-inductive sensor technology to record three-axis magnetic field variations with an adequate field resolution of less (or better) than 3 nT at a 1 Hz sample rate. The measurement range of the sensor is $\pm 1.1\times 10^{6}$ nT and is valid over a temperature range of −40 °C to +85 °C. Data from the PSWS network will combine these magnetometer measurements with high frequency (HF, 3–30 MHz) radio observations to monitor large-scale current systems and ionospheric disturbances due to drivers from both space and the atmosphere. A densely-spaced magnetometer array, once established, will demonstrate their space weather monitoring capability to an unprecedented spatial extent.

The primary goals are (1) to provide the general context of geomagnetic activity during the HF experiments proposed in the PSWS project; (2) to estimate ionospheric currents at mid/high latitudes; (3) to measure space weather-related disturbances (dB/dt) at higher latitudes.

HF communication is affected by the ionospheric conditions which are inarguably associated with the solar and geomagnetic activity. Magnetic field observations provide critical information about such an interaction (primary goal #1). As for primary goal #2, the proposed densely-spaced magnetometer network can also provide a complementary data set to help infer ionospheric currents over the entire region where the PSWS is located. At present, ground magnetometers for space research are located primarily near/in the auroral regions. The US government-funded magnetometers are sparsely located across the US at mid latitudes. The HamSCI magnetometer network will help increase the spatial resolution of the magnetic field measurements instead of taking on the existing infrastructure which is designed to provide well-calibrated absolute values of geomagnetic fields.

The higher spatial density is one of the major unique aspects of the PSWS project. Typically, ground magnetic field data are used to infer equivalent current systems in the ionosphere by combining the horizontal fields at multiple locations (e.g. [4], [5], [6]. At higher latitudes where more geomagnetic field lines are concentrated, a more densely-spaced sensor network is desired to increase the number of measurement points and thus to avoid interpolations based on an assumption that ionospheric conditions are uniform between the measurement points: we know that this is an oversimplification. While high-latitude (higher than the auroral region), multi-point magnetic fields from a densely-spaced sensor network have reported in a number of papers, mid-latitude observations of geomagnetic fields from a densely-spaced sensor network are much less. Space weather-related events (e.g., geomagnetically induced current or GIC, related to dB/dt) are also target observations, especially at high latitudes (e.g., Alaska) as stated in primary goal #3. Measurements of dB/dt with a higher spatial resolution have not been conducted as well.

The target specifications and performance level of the magnetometer are: (a) time-varying field measurements in three axes; (b) ∼3 nT resolution at 1 Hz sample rate; and (c) ∼50–100 mile spacing. Such a citizen-science level, large-scale, densely spaced magnetic field observations have never been done before. There are still many unsolved questions as to how the magnetosphere and ionosphere respond to solar activity, particularly, in greater details (in terms of spatial scale). During geomagnetic storms and substorms, magnetic field variations are on the order of tens to hundreds of nT, which are well within the observable range of magnetometers that we have developed.

## 2 Hardware description

The HamSCI magnetometers use an off-the-shelf, low-cost magnetic sensor (RM3100) using a new technology (“magneto-inductive”), manufactured by PNI Corp. The sensor is capable of measuring magnetic fields with a resolution of ∼3 nT at 1 Hz with a noise floor of 4 pT/$\sqrt{\mathrm{Hz}}$ at 1 Hz. A previous study [7] demonstrated that the sensor performance is adequate for both space-borne and ground-based applications to study geomagnetic activity. Given that the sensor will be located in various electromagnetic environments by amateur participants, we expect the performance to be somewhat compromised and thus our target level is ∼10 nT at 1 Hz.

**Fig. 1:**
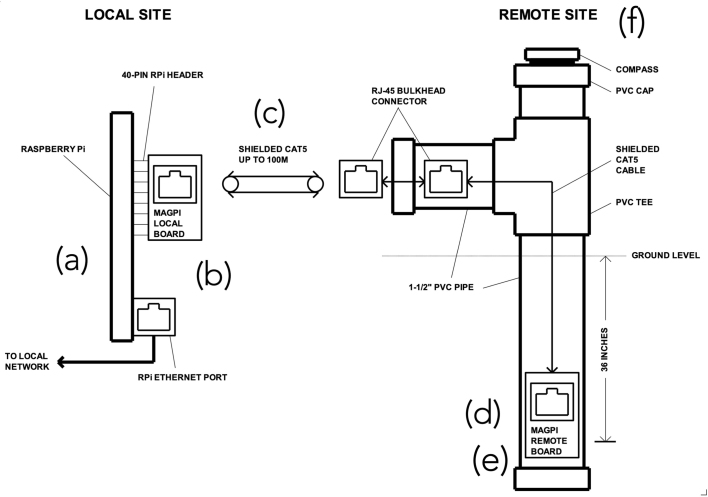
HamSCI magnetometer system diagram.

**Fig. 2:**
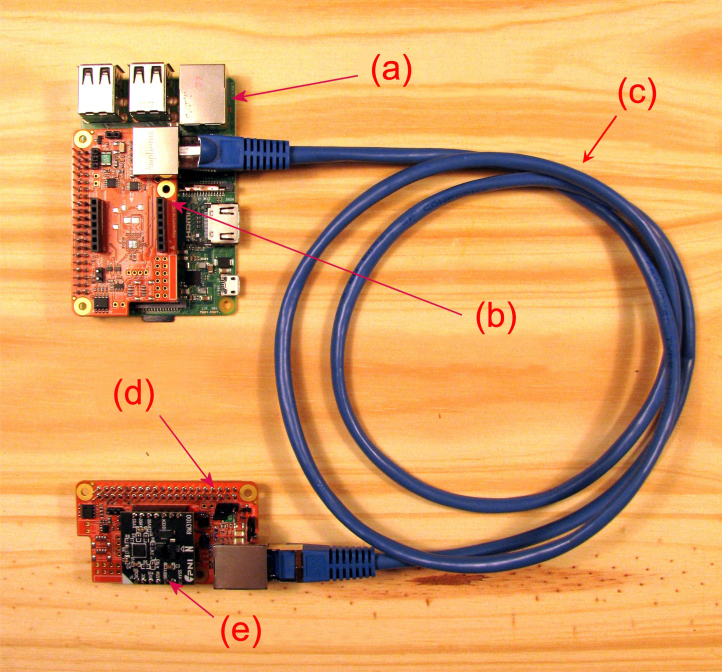
Assembled HamSCI magnetometer system (without the sensor burial kit).

As shown in the diagram in Fig. 1, the magnetometer system consists of five basic components: (a) local computer (i.e. Raspberry Pi); (b) local magnetometer support board extender; (c) shielded CAT5 interconnecting cable; (d) remote magnetometer support board extender; (e) PNI RM3100 magnetic sensor; and (f) magnetic sensor burial parts kit. The remote board (Item (d)) contains the RM3100 sensor which needs to be placed outside away from any sources of EMI while both the computer and local board are located inside. A recommended cable between the local and remote boards is a CAT5E or CAT6 A shielded twisted pair (solid copper, NOT high resistance copper coated aluminum (CCA)). The photo of the system is shown in Fig. 2). The magnetometer kit sold at the Tucson Amateur Packet Radio (TAPR) online store consists of the assembled and tested local and remote boards. The sensor burial parts kit is to be made by each user. The bill of material and design details for the sensor burial kit are available online (see Section 3. Design Files and Section 4. Bill of Materials).

The magnetometer package is expected to be approximately 250 US dollar (USD), which is much cheaper than any conventional magnetometers whose prices range between thousands and tens of thousands of USD. Again, the performance level of the magnetometer is lower (field resolution, in particular) than that of the high-performance, science-grade magnetometers deployed at geophysical observatories across the US. We note, however, that the proposed network is focused primarily on their spacing to increase the spatial resolution. Given the adequate resolution level (<10 nT at 1 Hz), most of the major geomagnetic activity (e.g., geomagnetic storms, substorms (may be limited to high-latitude regions) and large-scale ULF waves/disturbances) is expected to be observed, which will be compared with the HF measurements.

**Basic Function of the Magnetic Sensor**

The PNI RM3100 3-axis magneto-inductive magnetometer was designed with a monotonic response range of $\pm 8\times 10^{5}$ nT and a sensitivity of better than 13 nT (at a cycle count > 200). Linearity over a $\pm 2\times 10^{5}$ nT span is 0.5%. It is a complete evaluation board which only requires a 2.0 to 3.6 V supply at a current of <2 mA and either an i2c or SPI bus interface, a perfect match to a microcomputer such as the Raspberry Pi or Odroid.

All magnetic field sensors using a high permeability material as the sensing element depend on the fact that the flux created by the external field either adds to or subtracts from the flux created by a sense or control winding. The RM3100 magneto-inductive sensor, however, implements an LR oscillator in each axis in which a DC current is reversed every other cycle, resulting in a difference in period in each cycle pair, which depends upon the external field value.

A 50 MHz clock in the RM3100 control ASIC drives a counter which measures the period of each half cycle and then totalizes the difference over the chosen number of measurement cycles to derive a field value. The greater the number of cycles, the higher the resolution of the measurement. Four hundred (400) cycles is a good value to use for a resolution <10 nT. A simple sliding 60 s average, providing 1 min sample points or the more complex averaging method adopted by International Association of Geomagnetism and Aeronomy (IAGA) should provide 3 nT resolution.

**Magnetic Sensor Packaging**

**Fig. 3:**
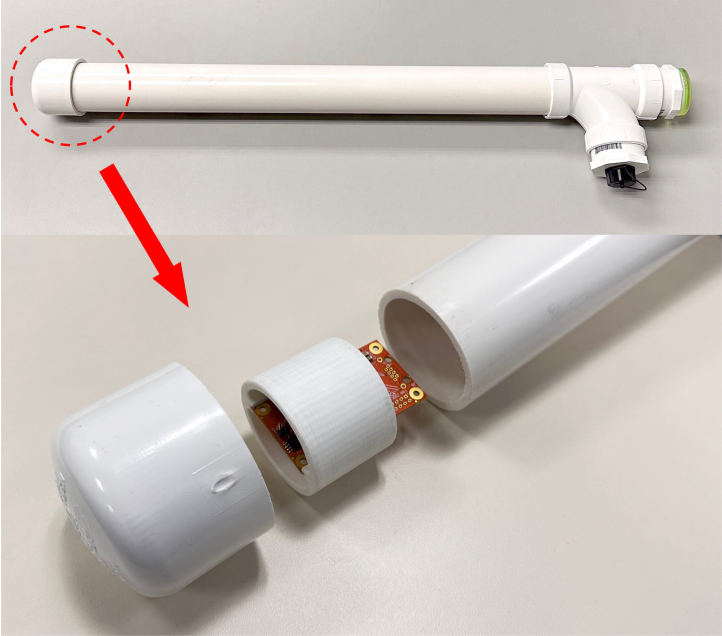
Magnetic sensor housing.

The sensor board (Item (d): remote magnetometer support board extender) is housed in a PVC pipe structure as shown in Fig. 3 for weather protection. It can be positioned vertically under the ground so that the sensor, located on the bottom of the pipe (the dashed circle in this figure), is well below the ground surface level, providing stable temperature. There is a 3-D printed structure that holds the remote board with the magnetic sensor in place as shown in the bottom of the figure.

## 3 Design files

All documentation and design files are available in editable form and archived in the open science framework, Zenedo at http://dx.doi.org/10.5281/zenodo.10289503. The information about the project can be found on the HamSCI web at https://hamsci.org/basic-project/personal-space-weather-station. All documentation is available as either Text (.txt) files, OpenDocument text files (.odt), or files in Comma Separated Values format (.csv). Other formats may be provided in addition in some cases. (GitHub Markdown (.md), and PDF). Design files have been created in KiCAD 1.x and are provided as complete files. The complete documentation collections are currently located at https://github.com/HamSCI/PSWS-Magnetometer. The design files are specific to the KiCAD 7.x development environment and of no practical concern to most people who might wish to replicate this work. Descriptions of the main file types by file extension can be found at: https://docs.kicad.org/7.0/en/kicad/kicad.html. The documentation is covered by the open source copyright requirements for CC by 4.0. Hardware is covered by the TAPR Open Hardware license. All supporting software is covered by the Gnu Public License 3.0. Table 1 summarizes the design files.

**Table 1:** Design files. All documentation and design files are available in editable form and archived in the open science framework, Zenedo at http://dx.doi.org/10.5281/zenodo.10289503.

Category	File	Description
Manual	TSDR-MAG-REQ-TangerineSDR-Magnetometer-V0.4.1.pdf	Magnetometer modules requirements document
	TSDR-MAG-ICD-TangerineSDR-Magnetometer-V0.1.1.pdf	interface control document
	MSB_Linux_Setup.pdf	A Linux setup instruction
	Software-Installation	A software installation guide for Raspberry Pi
	MagPi_QuickStart.pdf	A quick-start guide

Circuit	MAGNETOPIHAT_XE4.pdf	Schematics
Design	TSDR-MAG-8000-XE1-210730.pdf	PCB layer design

Sensor	RM3100 magnetometer assembly in PVC pipe-1.pdf	Sensor housing assembly instruction
Housing	RM3100 Sleeve.pdf	Sensor mount design schematic
	RM3100 Sleeve.STL	Sensor mount design file

Bill of material	rm3100PiHatLocal.csv	Local board part list
	rm3100Remote.csv	Remote board part list

## 4 Bill of materials

The bill of materials for construction of the magnetometer system is presented in Table 2. The magnetometer local and remote boards (with most of the electronic components soldered) along with the RM3100 sensor (named “Tangerine SDR Magnetometer”can be purchased from the TAPR online store (Items (b), (d), and (e)). Interested readers are advised to refer to the bills of materials (rm3100PiHatLocal.csv and rm3100Remote.csv) that list all the electronic components as shown in Table 1. The circuit design (schematics and PCB layout) is also included as part of the design files. The magnetic sensor (RM3100) housing (Fig. 3) is to be built with user-supplied components. The materials and design are provided in the design files as listed in Table 1. It is expected that users provide the computer peripherals such as monitor, keyboard, mouse, HDMI cable, etc.

**Table 2:** Bill of materials for the magnetic sensor and electronics.

Designator	Component	Number	Source	Unit cost	Total cost
Tangerine SDR Magnetometer	Magnetometer electronics	1	TAPR	$120	$120.00
Raspberry Pi3B with 1 GB memory	Single board computer	1	Amazon	$49.69	$49.69
Raspberry Pi3B case	Raspberry Pi3B case	1	Amazon	$9.69	$9.69
Raspberry Pi power supply	Power supply	1	Amazon	$19.99	$19.99
Micro SD Card (32 GB)	microSDHC Flash Memory Card	1	Amazon	$5.49	$5.49
CAT5E or CAT6A cable, outdoor, 100 ft	Ethernet cable	1	Amazon	$19.99	$19.99
				Total cost:	$ 224.85

## 5 Build instructions

As mentioned in the earlier sections, the magnetometer electronics (“local” and “remote” boards, Items (b) and (d) in Fig. 2, respectively) can be purchased from the TAPR online store and are mostly pre-assembled only with a few parts needed to be soldered by the users. The parts that need soldering include: (1) two (2) RJ-45 jacks; (2) one (1) 2 × 20 Pin, long-tail, female header; (3) two (2) 7-pin male pin headers; (4) two (2) 0.1” shorting jumpers; (5) one (1) PNI RM3100 magnetic sensor module. The 7-pin headers (Item #3) are to be soldered on the magnetic sensor module (Item #5) which is connected to the remote board (see (c) and (d) in Fig. 2). The local board ((b) in Fig. 2) is connected to the Raspberry Pi computer via the 2 × 20 pin header (see (a) and (b) in Fig. 2). The local and remote boards are connected via the Ethernet cable. The magnetic sensor module along with the remote board is housed in a PVC pipe for weather protection using user-supplied components (see Fig. 3. See the design files (Table 1) for detailed assembly instructions.

## 6 Operation instructions

**Data Acquisition Software**

The data acquisition software (“runMag”) should be installed on Raspberry-Pi to control the magnetometer system. This is a program intended to assist in testing the PNI RM3100 geomagnetic sensor. The RM3100 support boards were developed for use with the PSWS radio receivers. These board pairs report magnetic field strength as three independent vectors, from which a total field strength may be derived. They also report the temperature in the immediate environment of the remotely placed sensor and at the near end of the pair as a fraction of a degree C. They may also be used standalone with only a Pi or Pi clone board. Various pieces of software have been used to develop, test, and run these boards as part of the hardware suite or as standalone low-cost monitors of the Earth’s magnetic field.

At one time or another, testers and developers of these boards have used utilities included in the Raspberry Pi OS I2C tools package, ad-hoc python scripts, and purpose-built programs written in C for their work. All are possible. The runMag utility is written as a Linux command line program and takes all configuration parameters from its command line. runMag has some built-in documentation that displays the command line options As long as the Pi I2C kernel driver is activated (usually by configuring I2C I/O in the Raspberry Pi setup utility), no other library dependencies are required.

This software was written to be used on boards such as the Raspberry Pi 3/4, Odroid, Nvidia Nano and their kin. It has been tested, if not thoroughly, on many similar single board designs. runMag was written with the expectation that the host provides the de facto standard 40 pin IO bus of Raspberry Pi 3’s and their clones. The runMag repository may be found at: https://github.com/wittend/rm3100-runMag.git

**Installation and Alignment**

**Fig. 4:**
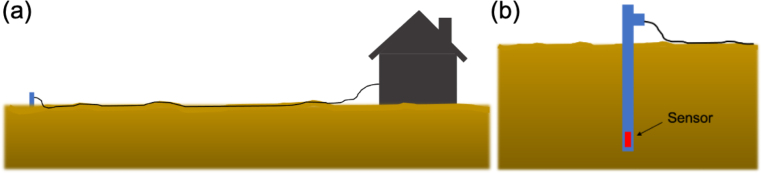
A sketch showing the sensor installation: (a) location of the sensor; (b) sensor housing.

**Fig. 5:**
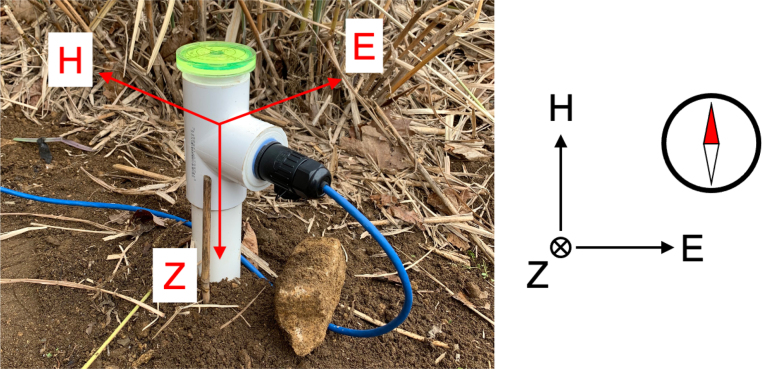
RM3100 sensor PVC pipe packaging installed on the ground.

The magnetometer system consists of the sensor and the electronics (the host computer) as described in the previous sections. The magnetic sensor should be located away from electromagnetic interference sources that may affect reliable measurements (e.g., magnetic objects, electronics, etc.) and be kept as constant a temperature as possible. The best practice is to locate the sensor package outside the building where the electronics is placed (Fig. 4). A minimum recommended cable length is 100 ft. In an area where EM noise is a concern, use a longer cable (depending on the nature of the noise source). CAT5E and CAT6 A can support up to 400 ft and 500 ft, respectively. The cable should be rated for direct burial. The PVC sensor housing is positioned vertically, leaving only the upper part of it (approximately 10 inches) above the ground surface as shown in Figs. 4(b) and 5. This provides temperature stability.

The sensor should be oriented in compliance with the widely accepted geomagnetic coordinate system, “HEZ”, in which H points toward the local geomagnetic north in the horizontal plane (perpendicular to the line pointing toward the center of the earth defined as Z). E completes the orthogonal system, pointing toward the geomagnetic east. Thus, Z=H × E in a vector form. Simply, H is defined as a direction which a compass needle would point toward. Fig. 5 shows the definition of the coordinate system and the sensor installation conforming to this. It should be noted that the sensor’s original XYZ coordinates do not match the HEZ coordinates because of how it is oriented in the pipe (the sensor was designed for use in the horizontal plane, but we are installing it in the vertical plane). Thus, a reallocation of coordinates is required in the post-processing data processing algorithm. That is, H =− Z, E=Y and Z=X where X, Y and Z are the original tri-axial components of the sensor output while H, E and Z are the local geomagnetic components that will be used for scientific use.

It is highly recommended that two persons work on the sensor orientation. The orientation of the sensor is done in the following order: (1) Bury the PVC housing in a vertical position, leaving only the upper part of it (approximately 10 inches) above the ground surface. Make sure the pipe is well leveled (using the bubble level on top of it) so it is vertical (i.e., aligned with the line toward the center of the earth) and can be rotated freely in the horizontal direction; (2) Find the direction of an approximate geomagnetic north, for example, using a compass and orient the pipe so the vertical line marked on the housing (see Fig. 3) points toward the geomagnetic north; (3) Turn on the magnetometer data acquisition system and run the acquisition software (refer to the operation software document provided separately); (4) While one person stays with the sensor outside, the other person monitors data output from the acquisition system. While monitoring the horizontal components of the data (H and E), rotate the pipe until the maximum positive value of H and near-zero value of E are obtained; (5) Repeat the procedure #4 until reliable horizontal values (H and E) are obtained. This step is particularly important during a geomagnetically disturbed time. Note that for geospace studies, magnetic field “variations” or “pulsations” are more important than absolute values of the fields. Thus, an extreme precision of sensor orientation is not required (as is done for magnetic field observations for geological or geophysical studies).

**Fig. 6:**
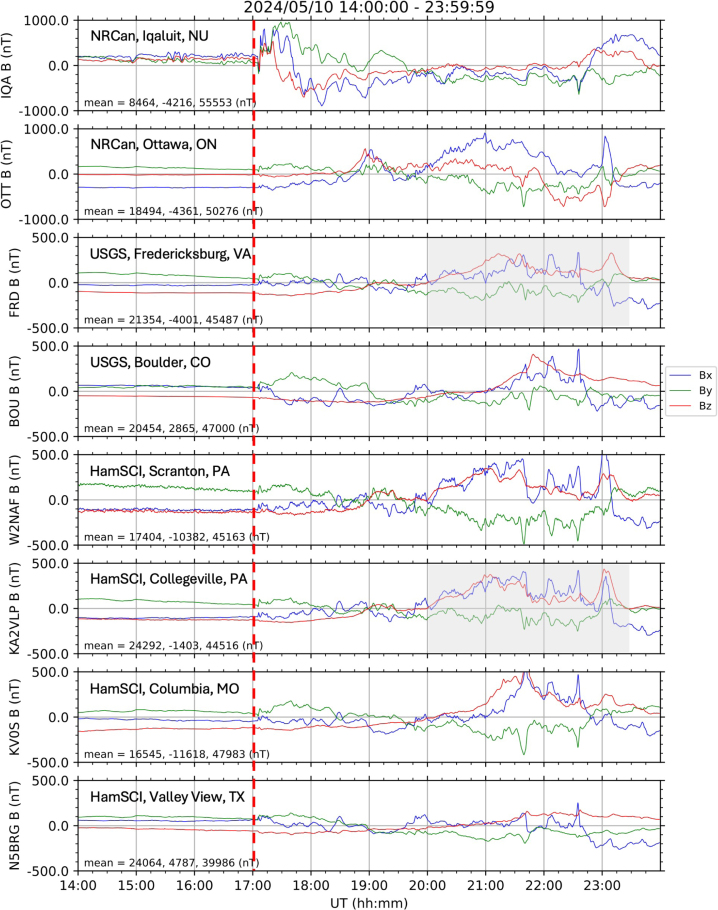
A geomagnetic storm (May 10, 2024, onset at around 17:00 UT as indicated by the red dashed line) observed by the RM3100 magnetometer systems installed at four locations (bottom four panels: Scranton, PA, Collegeville, PA, Columbia, MO and Valley View, TX) along with the professional science-grade magnetometers operated by US and Canadian agencies (top four panels: Iqaluit, NU, Ottawa, ON, Fredericksburg, VA and Boulder, CO). The panels are arranged in order of the geomagnetic latitudes of the stations. The mean values for each axis data (Bx, By and Bz, respectively) are displayed in each panel. Note that the Y-axis range for the data from Iqaluit and Ottawa (top two panels) is ±1000 nT while that for the other data is ±500 nT as the geomagnetic responses to solar wind activities are typically more intense and dynamic at higher latitudes.

**Fig. 7:**
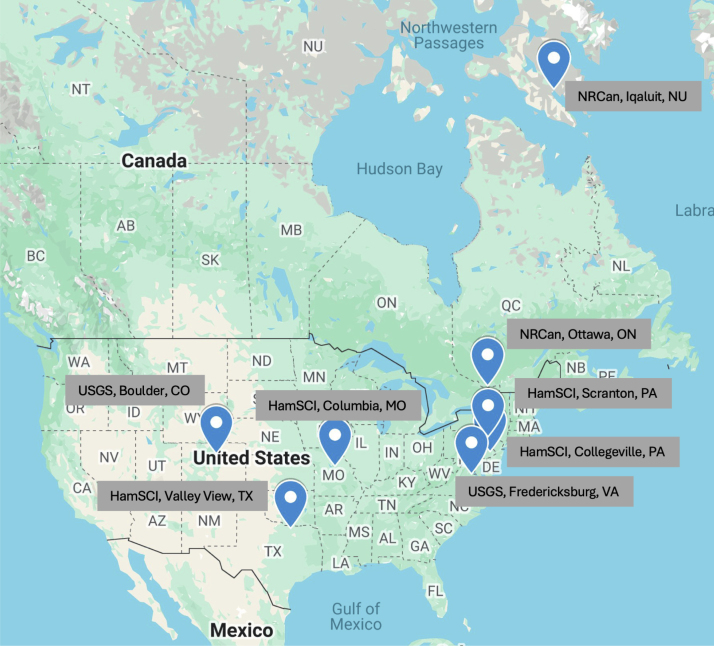
A map showing the locations of the stations used in Fig. 6.

**Fig. 8:**
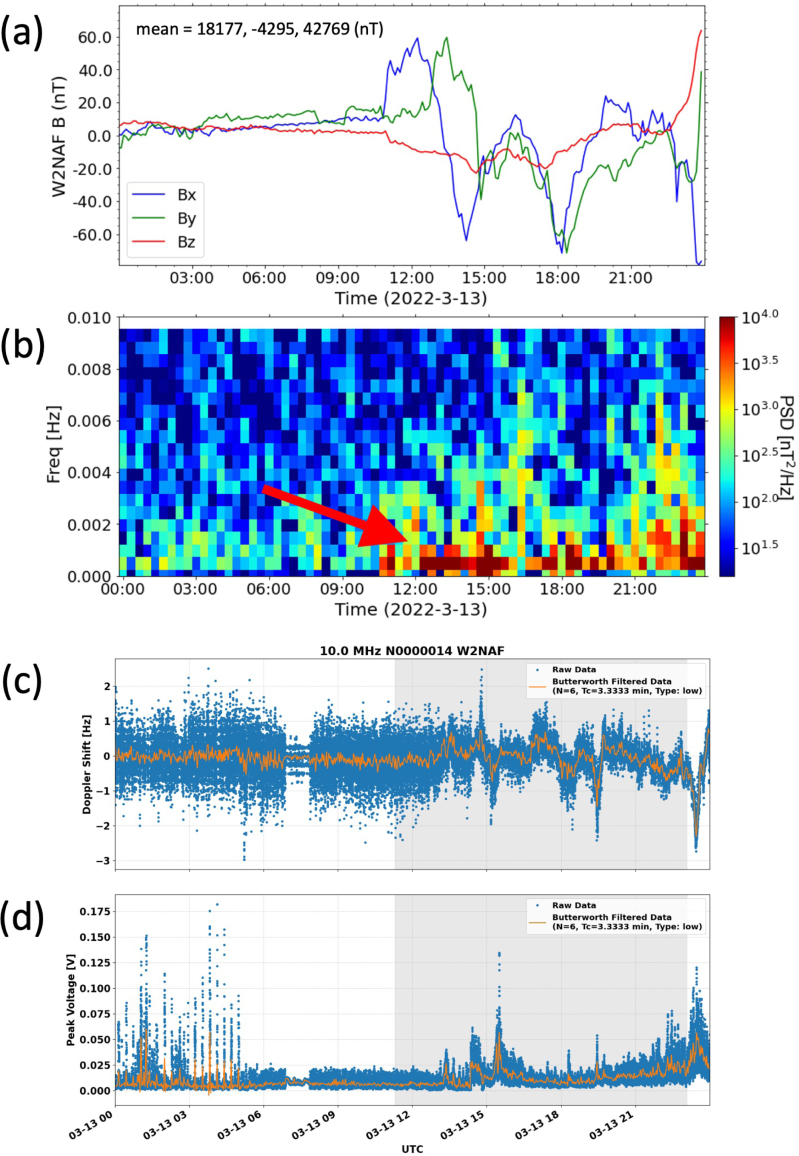
Geomagnetic pulsations during a geomagnetic storm on March 13, 2022. The time series plot (Panel (a)) and fast Fourier transform (FFT) spectrogram showing geomagnetic pulsations (near 2 to 7 mHz) are clearly shown. The mean values for each axis data (Bx, By and Bz, respectively) are displayed in Panel (a). Doppler shifts (Panel (c) and amplitude variations (Panel (d)) in the HF signals received by the co-located GRAPE HF receiver were simultaneously measured.

## 7 Validation and characterization

Once installed, the system can be verified by comparing its data with the predicted values from a model called International Geomagnetic Reference Field (IGRF), an approximate model field at a particular location [8]. Approximate values for a specific year can be obtained from web-based tools provided by NASA (“OMNIWeb” at https://omniweb.gsfc.nasa.gov/vitmo/cgm.html) or Kyoto University World Data Center for Geomagnetism ( https://wdc.kugi.kyoto-u.ac.jp/igrf/point/index.html) which provide model magnetic fields at a specific geographic location. Users only need the sensor location in geographic coordinates (latitude/longitude and altitude) and year of interest. Each component can be compared. However, it is important to note that the northward (X) and eastward (Y) in the IGRF are different from the H and E coordinates as the IGRF reports “geographic” north and east whereas our system is oriented with respect to the geomagnetic north and east. Thus these two values should not be compared directly. They are related by X=Hcos(D) where D is declination, the angle on the horizontal plane between magnetic north and true north. Nevertheless, the total horizontal field should be close to each other: that is, horizontal field $=\sqrt{X^{2}+Y^{2}}=\sqrt{H^{2}+E^{2}}$. The downward components in both coordinate systems (Z) are identical. In addition, the total field can be compared to see if they are similar, as defined as $\sqrt{X^{2}+Y^{2}+Z^{2}}=\sqrt{H^{2}+E^{2}+Z^{2}}$. Note this is labeled as “F” (total intensity) on the IGRF website. The IGRF values are only from a model and thus it is natural that there are some discrepancies between the actual measurements and the IGRF values. This can be more extreme at high latitudes and during geomagnetically disturbed times.

Presented in Fig. 6 are the example data during the geomagnetic storm on May 10, 2024, which is considered as the most powerful geomagnetic storm in recent years, obtained by the HamSCI magnetometer systems installed at four locations (bottom four panels: Hope, NJ, Scranton, PA and Columbia, MO) along with the data from the professional science-grade magnetometers operated by US and Canadian agencies (top four panels). The locations of the stations are shown in the map in Fig. 7. It is clear that the HamSCI magnetometers measured geomagnetic variations during the geomagnetic storm event as well as the observatory magnetometers did. The storm began at ∼17:00 UT (as indicated by the red dashed line). Due to their locations (in different geomagnetic latitudes and longitudes), the magnetic field traces may be slightly different. However, the HamSCI magnetometers detected the field variations characterizing geomagnetic storm activities such as the storm onset at ∼17:00 UT and transient changes (shown as sudden increases/decreases in the field variations) displaying a similar deflection range of up to 500 nT. In particular, the magnetic field variations measured from the Fredericksburg (USGS) and Collegeville (HamSCI) stations are quite comparable as they are close both geographically and geomagnetically (see the gray shaded boxes in Fig. 6).

As mentioned earlier, for the purposes of geospace investigations where absolute value measurements (B) are not very critical but rather variations (dB), the data shown in Fig. 6 are background-removed (i.e., dB = original data (B) - mean(B)). In addition, since the frequency range of interest is in the ULF range (tens of seconds to tens of minutes or a few mHz), the HamSCI magnetometer data have been moving-averaged with a 60-s window to filter out higher frequency uncertainty to decrease the noise level in the higher frequency range. The same technique has been implemented by [7].

Fig. 8 demonstrates geomagnetic pulsations measured by the HamSCI magnetometer during a geomagnetic storm occurred on March 13, 2022. The time-series data (Panel (a)) clearly shows the compression of the magnetosphere (∼10:30 UT) due to an increased solar wind pressure (combination of solar wind velocity and density) followed by an increased currents due to plasma particles around the Earth. The fast Fourier transformed (FFT) spectrogram (Panel (b)) displays band-limited ULF wave events (near 2 to 7 mHz) associated with the geomagnetic storm. The panels (c) and (d) in this figure show the Doppler shifts and amplitude variations in the HF signals received by the co-located GRAPE HF receiver, respectively. The Doppler shift indicates ionospheric disturbances during the geomagnetic storm event.

The example events shown above clearly demonstrate the adequate level performance of the amateur-grade magnetometer systems. There are, of course, limitations in terms of its performance (e.g., field resolution) compared to the much more expensive science-grade magnetometers which can measure very subtle magnetic field variations. Though these types of variations are scientifically important, the target of the present project is mainly to monitor large-scale variations that concerns the space weather that may impact radio operations.

## 8 Data access

For data dissemination and archival, data from each location will be collected and stored in a central data portal through which users can view and download acquired data. The HamSCI group is currently developing a server system that combines the magnetometer and HF radio receiver data to support the broader community’s scientific involvement. While this is being developed, the acquired magnetometer data are publicly available at https://picomation.net/.

## CRediT authorship contribution statement

**Hyomin Kim:** Writing – review & editing, Writing – original draft, Visualization, Validation, Supervision. **David Witten:** Validation, Software. **Julius Madey:** Validation, Resources, Methodology. **Nathaniel Frissell:** Project administration, Funding acquisition, Conceptualization. **John Gibbons:** Validation. **William Engelke:** Visualization. **Anderson Liddle:** Visualization. **Nicholas Muscolino:** Visualization. **Joseph Visone:** Validation. **Zhaoshu Cao:** Visualization.

## Declaration of competing interest

The authors declare that they have no known competing financial interests or personal relationships that could have appeared to influence the work reported in this paper.
